# Simulating forest resilience: A review

**DOI:** 10.1111/geb.13197

**Published:** 2020-10-08

**Authors:** Katharina Albrich, Werner Rammer, Monica G. Turner, Zak Ratajczak, Kristin H. Braziunas, Winslow D. Hansen, Rupert Seidl

**Affiliations:** ^1^ Institute of Silviculture University of Natural Resources and Life Sciences (BOKU) Vienna Wien Austria; ^2^ Ecosystem Dynamics and Forest Management Group Technical University of Munich Freising Germany; ^3^ Department of Integrative Biology University of Wisconsin‐Madison Madison Wisconsin USA; ^4^ Earth Institute Columbia University New York City New York USA

**Keywords:** ecosystem modelling, literature review, model development, process‐based modelling, resilience processes, simulation model

## Abstract

**Aim:**

Simulation models are important tools for quantifying the resilience (i.e., persistence under changed environmental conditions) of forest ecosystems to global change. We synthesized the modelling literature on forest resilience, summarizing common models and applications in resilience research, and scrutinizing the implementation of important resilience mechanisms in these models. Models applied to assess resilience are highly diverse, and our goal was to assess how well they account for important resilience mechanisms identified in experimental and empirical research.

**Location:**

Global.

**Time period:**

1994 to 2019.

**Major taxa studied:**

Trees.

**Methods:**

We reviewed the forest resilience literature using online databases, selecting 119 simulation modelling studies for further analysis. We identified a set of resilience mechanisms from the general resilience literature and analysed models for their representation of these mechanisms. Analyses were grouped by investigated drivers (resilience to what) and responses (resilience of what), as well as by the type of model being used.

**Results:**

Models used to study forest resilience varied widely, from analytical approaches to complex landscape simulators. The most commonly addressed questions were associated with resilience of forest cover to fire. Important resilience mechanisms pertaining to regeneration, soil processes, and disturbance legacies were explicitly simulated in only 34 to 46% of the model applications.

**Main conclusions:**

We found a large gap between processes identified as underpinning forest resilience in the theoretical and empirical literature, and those represented in models used to assess forest resilience. Contemporary forest models developed for other goals may be poorly suited for studying forest resilience during an era of accelerating change. Our results highlight the need for a new wave of model development to enhance understanding of and management for resilient forests.

## 1 INTRODUCTION

Forest ecosystems are under increasing pressure from changing environmental drivers and intensifying disturbances related primarily to changes in climate and land use (McDowell et al., 2020; Millar et al., 2007; Scholze et al., 2006; Trumbore et al., 2015). These changes can move ecosystems out of their historical range of variability (Keane et al., 2009), possibly causing unexpected and nonlinear responses, such as abrupt transitions to other ecosystem states (Albrich et al., 2020; Ratajczak et al., 2018). This uncertainty in future ecosystem trajectories presents challenges for ecosystem managers tasked with ensuring that ecosystems will be able to cope with these changes. It is also difficult for researchers to investigate responses to conditions for which no historical analogues exist.

The concept of resilience provides a framework for assessing the response of ecosystems to changing pressures. Resilient forests, that is, those that are able to persist even under changed environmental conditions, are frequently mentioned as a main goal of forest management and restoration (Bone et al., 2016; Keenan, 2015; Rist & Moen, 2013; Seidl et al., 2016). Despite a wide adoption of resilience in applied ecology its specific meaning often remains unclear, as resilience is difficult to operationalize and quantify (Carpenter et al., 2001; Standish et al., 2014). Resilience has many definitions (Box 1; Brand & Jax, 2007; Grimm & Wissel, 1997), but in ecology, resilience is most often used to describe the response of ecosystems to disturbances or other changes in environmental drivers. Resilience here is the ability to maintain a functionally similar state despite changes in disturbances and other drivers, by being resistant or ‘bouncing back’ when the system drifts from its long‐term state (Walker et al., 2004).

Box 1 **Resilience definitions**

While many different definitions of resilience exist, the following three are most frequently used in forest ecosystem research (see Nikinmaa et al., 2020). Further considerations of the resilience concept and its definitions can be found in, for example, Grimm and Wissel (1997), Carpenter et al. (2001), Brand and Jax (2007) and Folke (2006).

**Engineering resilience** (Pimm, 1984) refers to the time a system needs to recover from a disturbance. It assumes the presence of a single equilibrium state that a system deterministically returns to after a disturbance.

**Ecological resilience** (Holling, 1973; Holling & Gunderson, 2002) is defined as the ability of a system to maintain its functions, structures and feedbacks in the face of disturbance. It acknowledges the presence of multiple equilibrium states, and the possibility that a system will not return to its state prior to disturbance but rather shifts to an alternative state.

**Socio‐ecological resilience** (Walker et al., 2004): focuses on coupled human and natural systems and their ability to stay within a desirable regime (i.e., maintain structures, functions and services) under disturbance. It particularly emphasizes the role of adaptation.

Assessing the impacts of environmental change is particularly challenging in forest ecosystems, due to their longevity and often protracted responses to change (Standish et al., 2014). Unlike faster systems, such as lakes, where experimental manipulations are routinely used to investigate ecosystem resilience (Schröder et al., 2005), experimental investigations of resilience are difficult in forest ecosystems due to the large time spans and spatial extents that are necessary to obtain inference. These challenges related to space and time make simulation models an important tool in forest resilience research. Models allow the impact of environmental changes that lack past analogues to be investigated. Furthermore, they enable experimentation in silico to assess recovery and collapse over larger spatial extents and temporal durations than would be possible through experimental manipulation (Egli et al., 2018; Seidl et al., 2016). A particular strength of simulation modelling in forest resilience research lies in its ability to consider multiple drivers simultaneously and to quantify their interacting impacts on forest ecosystems. Models allow for a more thorough exploration of these impacts on state variables (e.g., forest cover, biomass) and the potentially large state spaces occurring in nature, enabling the identification of alternative system states.

A comprehensive picture of how simulation models are used in the context of forest resilience, and how important processes are implemented in these models, is lacking to date. Recent years have brought an improved understanding of processes that contribute to forest resilience (e.g., legacies, forest regeneration processes, Johnstone et al., 2016), but model development often lags behind this understanding, meaning that crucial processes of forest resilience may not yet be included in models.

An improved synthetic understanding of the models used to assess resilience could further forest research in at least three important ways: First, it allows researchers aiming to study resilience to identify promising modelling approaches. Second, it can identify novel modelling approaches that have not yet been applied widely to questions of resilience. And third, the identification of resilience mechanisms that have received only limited attention in models could stimulate the development of improved models for simulating resilience. Here, our aim was to provide a review and synthesis of the simulation models used to study forest resilience. Specifically, our objectives were to synthesize (a) which questions of resilience are addressed with simulation models, (b) what types of models are used for specific resilience questions, and (c) whether processes identified as important for resilience in the theoretical/empirical literature are represented in simulation models.

## 2 METHODS

### 2.1 Definition of resilience

A necessary first step in conducting our review was to operationalize our definition of resilience, enabling us to identify relevant studies. Resilience is a frequently used term with an evolving set of definitions (Brand & Jax, 2007; Nikinmaa et al., 2020; Ratajczak et al., 2018). We chose an approach suggested by Carpenter et al. (2001), operationalizing resilience by assessing the *resilience of what* (i.e., which forest ecosystem property responds) and the *resilience to what* (i.e., the pressure or driver that triggers a response). This allowed us to compare studies that themselves used very different definitions of resilience, for example, from engineering resilience (i.e., the ability of the system to resist disturbance and the rate at which it returns to equilibrium after a disturbance, as defined by Pimm, 1984; applied e.g., in Seidl et al., 2017) to socio‐ecological resilience (i.e., the capacity of a coupled human‐natural system to absorb disturbances and maintain its essential functions, processes, and feedbacks, as defined in Adger, 2005 and Walker et al., 2004; applied e.g., in Charnley et al., 2017).

### 2.2 Literature search and identification of relevant studies

To identify relevant studies in the scientific literature, we conducted an extensive web search using the academic literature databases Scopus (Elsevier, Amsterdam, Netherlands) and ISI Web of Science (Clarivate Analytics, Philadelphia, PA, USA). We searched for the terms “forest” and “model*” in combination with any of the terms “resilien*”, “state shift”, “regime shift”, “tipping point”, “recovery rate”, “catastrophic shift”, “abrupt shift”, “bifurcation” “bistab*” or “collapse” (search terms based on and expanded from Ratajczak et al., 2018). We included only studies published in English. The cut‐off date for publications to be included in this study was 6 September 2019.

The search yielded more than 1,200 entries, which were manually checked to filter studies that were of relevance for our research questions. Specifically, we checked whether the study investigated forest ecosystems (e.g., we did not include studies that focused on transitions between grassland and savanna ecosystems, where forest was not one of the possible ecosystem states), used some sort of simulation model (studies using purely conceptual models without numerical simulation, or that consisted of fitting a statistical model to data were not included), and investigated resilience (omitting studies that mentioned the term in the abstract or keywords but whose study objectives were not related to resilience). This selection resulted in a total of 119 studies being included in our review, representing 128 individual model applications (as a few studies included multiple models).

From each study we collected information for several categories (Table 1). General information such as location of the study and the investigated biome and ecosystem type allowed us to identify geographical ‘hotspots’ of simulation model use in resilience research. We also recorded the name and type of model as well as a set of essential model characteristics (spatial and temporal grain and extent, spatial explicitness, stochasticity, and whether the model was process‐based) to better characterize the types of models that are used for simulating forest resilience. We also investigated how resilience was defined in each study, specifically noting the *of what* and *to what* (sensu Carpenter et al., 2001), and recording specific response variables used to quantify resilience where applicable (this includes both dedicated resilience indicators, as defined for example by Scheffer et al., 2015, and relevant measurements of state variables, e.g., biomass, species shares). The responses (*of what*) and drivers (*to what*) were recorded jointly so that relevant response/driver combinations could be identified. As drivers often do not act in isolation, the co‐occurrence of different drivers was also analysed.

**TABLE 1:** Information gathered from each simulation study of forest resilience

Category	Subcategory	Indicator	Information recorded
General information		Location	As reported by the authors
		Biome	According to categories of Olson et al. (2001)
		Forest ecosystem type	As reported by the authors
Model information		Model type	Categories: analytical/conceptual, biogeochemical, dynamic global vegetation model (DGVM), empirical, landscape, population, state & transition, other
		Spatial explicitness	Is the model spatially explicit?
		Spatial grain and extent	In hectares, pixel size and size of simulated area
		Temporal grain and extent	In years, smallest time step and simulation duration
		Stochasticity	Are any stochastic processes implemented?
		Process‐based	Is the model process‐based?
Resilience definition		Of what	Categories: forest cover, forest composition, forest structure, forest functioning, ecosystem services, biodiversity
		To what	Categories: climate change, land use, fire, drought, wind, other abiotic, insect, other biotic, generic (no agent given), other (fits none of the above categories)
		Definition	Which definition of resilience do the authors give, if any?
		Quantification	How is resilience quantified?
Resilience processes	Regeneration: are processes related to regeneration implemented in the model?	Reproductive maturity	Do trees have to reach maturity before they can reproduce?
		Serotiny	Is the process of serotiny (regarding seed availability after fire) implemented in the model?
		Resprouting	Are trees able to resprout in the model?
		Distance to seed source	Is spatial dispersal of seeds considered in the model?
		Climate sensitivity	Is regeneration sensitive to climate influence?
		Competition from other vegetation	Is regeneration sensitive to competition from other vegetation (adult trees, herbaceous vegetation)?
		Light availability	Is regeneration sensitive to light availability (influence of canopy layer)?
	Legacy processes: is tree survival and the carryover of information as well as material legacies in the face of disturbance simulated?	Disturbance tolerance	Do live trees remain behind after disturbance?
		Maturity effect on disturbance tolerance	Does the age/size of trees (adult tree versus sapling) influence their environmental response/susceptibility to disturbance?
		Seed bank	Are seed banks (aerial and soil) implemented in the model?
		Persistent stress	Does the model track the influence of stress on tree survival over multiple time steps?
	Soil processes: are soil processes included in the model?	Water availability	Is water availability (soil moisture) a factor influencing forests in the model?
		Erosion	Can erosion (loss of fertile soil) happen in the model?
		Nutrient cycling (nitrogen and other nutrients)	Does the model include dynamic nutrient cycles (for nitrogen and other nutrients)?

### 2.3 Analysis of process inclusion

The core of our review consists of the analysis of specific ecological processes deemed important for forest resilience and their implementation in models. This catalogue of processes was compiled a priori, and is based on seminal work on forest resilience (Frelich & Reich, 1999; Johnstone et al., 2016; Martínez‐Vilalta & Lloret, 2016). We identified three groups of processes of particular relevance for forest resilience: regeneration processes, legacy processes and soil processes. We purposefully kept the set of processes investigated general as we acknowledge that model formulations necessarily vary with different study systems.

Regeneration is a crucial contributor to forest resilience as it strongly influences post‐disturbance recovery. Regeneration processes have a large influence on whether the ecosystem is able to recover, or whether it shifts to a different type of forest or a non‐forest state (Enright et al., 2014; Johnstone et al., 2016; Martínez‐Vilalta & Lloret, 2016). Specifically, we investigated whether *distance to seed source*, *reproductive maturity, serotiny,* and *resprouting* – processes related to the availability of reproductive material (seeds and sprouts) – were considered in the models. We also considered the *climate sensitivity of regeneration*, *light availability* (shading effects from mature trees) and *competition from other (non‐tree) vegetation*, as these processes often have a strong bearing on the survival of seedlings and saplings.

Legacy processes are mechanisms that lead to information or material being carried over from the pre‐disturbance ecosystem into the post‐disturbance ecosystem (Johnstone et al., 2016). They are often directly related to regeneration processes, as they can provide starting points for recovery, for example in the case of *aerial and soil seed banks*. We also analysed *live tree legacies*, which are a measure of tree *tolerance to disturbance* and represent an important seed source that is carried over from the pre‐disturbance state of the ecosystem (Seidl et al., 2014). Finally, we investigated *persistent stress* as a legacy, that is, whether the model tracks the influence of stress on tree survival over multiple time steps (Anderegg et al., 2015; McDowell et al., 2008).

Soil processes identified as relevant for ecological resilience were primarily related to water and nutrient availability (Fahey et al., 2016; Gazol et al., 2017; von Oheimb et al., 2014). We therefore assessed the implementation of *water availability* and *nutrient cycling* (separately for nitrogen and other nutrients) in the models used to investigate forest resilience. We also hypothesized that *soil erosion* is important for resilience (Flores et al., 2019), and analysed its implementation in the models applied in the resilience literature. All data analysis and visualization was conducted in R (version 3.6.2, R Core Team, 2019), specifically using the packages dplyr (version 0.8.3, Wickham et al., 2019) ggplot2 (version 3.2.1, Wickham, 2016), reshape2 (version 1.4.3, Wickham, 2007) and networkD3 (version 0.4, Allaire et al., 2017).

## 3 RESULTS

### 3.1 Use of forest resilience and the geography of its application

**FIGURE 1:**
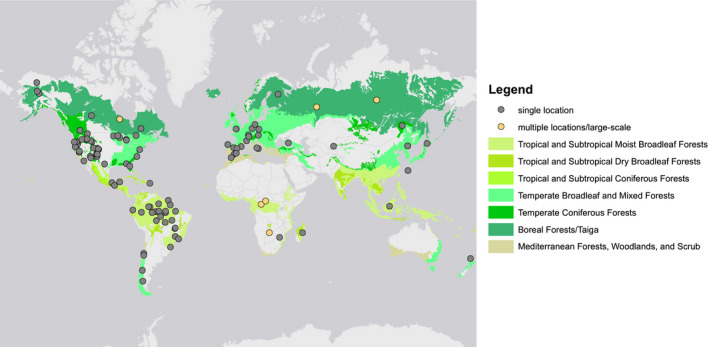
Geographical distribution of simulation studies addressing forest resilience. Each dot represents one study. For studies that covered a large spatial extent or included multiple study sites the location is given as the centre point of the area addressed. In addition to the 86 georeferenced studies displayed here our analyses also included 33 studies that had no specified location or were global in their extent. (Biome map from Olson et al., 2001.)

The use of resilience as a concept in forest research has increased over time (see Supporting Information Material S1: Figure S1.1). Most of the studies included in this analysis focused on forest resilience in the Americas (North and South) or Europe (Figure 1). The tropical and temperate biomes were particularly well represented. Most studies simulating resilience focused on a single study site or a small number of study locations. However there were also studies covering larger (e.g., sub‐continental) areas and many different study sites (e.g., Shuman et al., 2011). Of the 119 studies investigated, 31 (26%) did not specify a location (i.e., the model was built for a certain biome or type of ecosystem but was not linked to a particular study site or landscape) and 2 (1.7%) were global in extent.

### 3.2 Drivers and responses in modelling forest resilience

**FIGURE 2:**
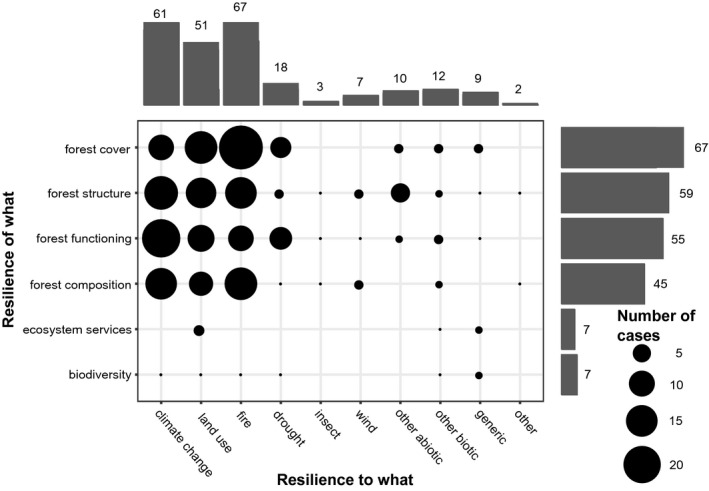
Combinations of responses (‘of what’, *y* axis) and drivers (‘to what’, *x* axis) in simulation studies of forest resilience

We grouped the studies by the responses (*of what*) and drivers (*to what*) that they addressed. There were 43 unique combinations of drivers and responses, with many studies investigating multiple drivers and responses. The most frequent response variables considered in model‐based forest resilience studies were, in order of decreasing frequency, forest cover, forest structure (referring to, e.g., tree size distribution), forest functioning (e.g., primary productivity) and forest composition (e.g., species occurrence and abundance, Figure 2). Overall, metrics of forest structure, function and composition were investigated more frequently compared to indicators linked to these ecosystem responses, such as variables associated with biodiversity and ecosystem services.

The most common drivers (*to what*) assessed in modelling studies were wildfire, climate change and land use (Figure 2). With the exception of fire, drivers related to human activity (such as land use and climate change) were more frequently investigated than natural disturbances (e.g., wind or insect disturbance). The two most commonly simulated drivers (climate change and fire) are also the ones most frequently considered together (Supporting Information Material S1: Figure S1.2). Overall, the most common driver‐response combination was fire and forest cover, followed by climate change and forest functioning (Figure 2).

### 3.3 What types of models are used to simulate resilience?

**FIGURE 3:**
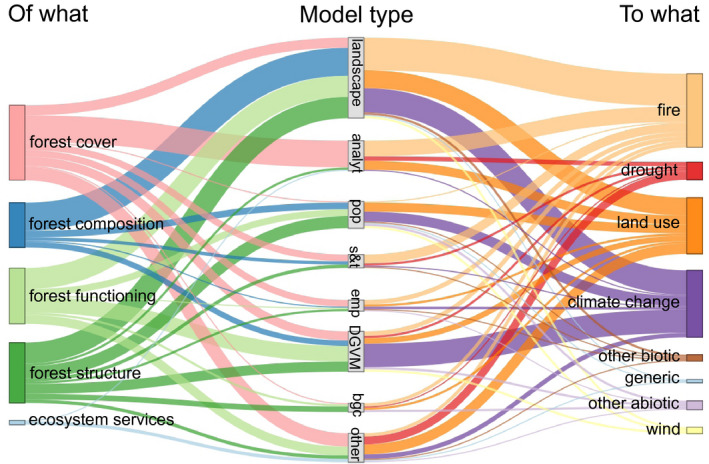
Model types used to simulate important response variables (resilience of what) to prominent drivers (resilience to what). Width of bands and bars is proportional to the number of studies found in the literature. For clarity only combinations that had more than three occurrences are shown here. Abbreviations: analyt = analytical/conceptual models; pop = population models; s&t = state and transition models; bgc = biogeochemical models; emp = empirical models; DGVM = dynamic global vegetation models.

The most common model type was landscape models. Analytical models (mostly consisting of a set of ordinary differential equations or similar), dynamic global vegetation models (DGVMs) and population models were also frequently applied to study forest resilience (Figure 3 and Table 2). However, several models could not be clearly classified into one of these broad model types. These include instances of coupling different types of models, for example, a DGVM linked to a state and transition model (Halofsky et al., 2014).

**TABLE 2:** Basic characteristics of the models used to simulate forest resilience

Model type	*n*	Spatial		Temporal	
		Grain (ha)	Extent (ha)	Grain	Extent (years)
Landscape	27	10^–1^	10^5^	Year	250
		4∙10^–4^ to 8∙10^1^	1∙10^0^ to 2∙10^7^	Day to decade	55 to 3,348
Conceptual/analytical	22	10^5^	10^8^	Year	500
		2∙10^1^ to 6∙10^5^	4∙10^7^ to 1∙10^9^	NA	90 to 10,000
DGVM	19	10^5^	10^7^	Day	100
		5∙10^–2^ to 3∙10^8^	1∙10^2^ to 3∙10^9^	Day to year	91 to 333
Population	19	10^–2^	10^1^	Year	500
		2∙10^–3^ to 1∙10^2^	1∙10^0^ to 3∙10^3^	Day to decade	5 to 1,575
Biogeochemical	9	10^6^	10^8^	Day	180
		1∙10^5^ to 1∙10^6^	5∙10^7^ to 10^9^	Day to year	4 to 8,096
State and transition	8	10^0^	10^6^	Year	150
		1∙10^–1^ to 4∙10^1^	5∙10^2^ to 6∙10^6^	NA	50 to 500
Empirical	6	10^0^	10^4^	Year	75
		1∙10^–2^ to 3∙10^5^	1∙10^3^ to 3∙10^8^	Year to decade	15 to 4,000
Other	18	10^–2^	10^4^	Year	90
		7∙10^–4^ to 1∙10^1^	1∙10^0^ to 8∙10^7^	Day to year	6 to 1,850

Note

DGVM = dynamic global vegetation model. Shown are number of observations (model applications) per model type, median, and 5th–95th percentile range for spatial grain and extent as well as temporal extent of model applications. For temporal grain (time step), the most common value and the highest and lowest resolution are shown.

Simulations of resilience were conducted over a wide variety of spatial grains (10^–4^–10^8^ ha) and extents (10^0^–10^9^ ha, Table 2). The simulated extent ranged from plot‐level to global simulations. There was similar diversity in temporal grain and extent. Most models simulating resilience operate on a yearly time step. While the simulation duration (temporal extent) varied enormously from a few years to several thousands of years, most studies covered a study period of less than 200 years (Supporting Information Material S1: Figure S1.3).

Certain model types were preferentially used to address specific drivers and responses (Figure 3). The most frequent response variable (forest cover) was most commonly addressed by simple conceptual models. The resilience of forest composition, forest functioning and forest structure was most often simulated with landscape models. DGVMs were frequently used to model the resilience of forest functioning. Landscape models and DGVMs are also important tools to assess forest resilience to climate change. With regard to resilience to fire, mainly landscape and analytical models were employed, while the effects of land use and climate change were addressed by several different types of models.

### 3.4 Implementation of resilience processes in simulation models

Overall, 67% of the model‐based studies investigating forest resilience were process‐based models, of which 41% were spatially explicit. Furthermore, 42% of models included thresholds and 41% included feedbacks. 96% of models included a representation of at least one process from our resilience processes catalogue.

#### 3.4.1 Regeneration processes

Regeneration processes are of high relevance for forest resilience. Yet, only 41% of the model applications considered regeneration processes explicitly (Table 3). Most of the model applications addressing regeneration explicitly considered the effect of reproductive maturity as well as the influence of light availability and climate on seedling survival and regeneration success. In contrast, the influences of competition from ground vegetation or herbivory (Supporting Information Material S3) were rarely considered. Also, only 17% of models simulate regeneration as an emergent property of multiple processes, such as the interplay of dispersal, establishment, and seedling growth. The level of detail with which regeneration was considered in models generally varied with the objective of the study (i.e., different of what/ to what combinations, Table 3).

**TABLE 3:** The explicit consideration of regeneration processes in models used to simulate forest resilience, parsed by different combinations of resilience of what and resilience to what for the most commonly occurring combinations

		n	Natural regeneration included (%)	Regeneration processes						
				Reproductive maturity (%)	Serotiny (%)	Re‐sprouting (%)	Distance to seed source (%)	Climate (%)	Competition (%)	Light availability (%)
All model applications		128	*41.4*	32.0	9.4	24.2	18.8	25.8	9.3	31.3
**Of what**	**To what**									
Forest functioning	Climate change	21	47.6	38.1	14.3	28.6	23.8	42.9	4.8	28.6
	Fire	9	77.8	66.7	33.3	66.6	66.7	77.7	0.0	66.6
	Land use	10	40.0	40.0	30.0	40.0	20.0	60.0	20.0	50.0
Forest structure	Climate change	16	62.5	50.0	25	37.5	25.0	37.5	6.3	50.0
	Fire	14	35.7	42.9	21.4	35.7	28.6	42.9	0.0	42.9
	Land use	13	76.9	61.5	23.1	61.5	23.1	46.2	7.7	69.2
Forest composition	Climate change	14	71.4	64.3	35.7	50	42.9	64.3	0.0	57.1
	Fire	15	86.7	73.3	26.7	66.7	60.0	60.0	6.7	53.3
	Land use	8	100.0	75.0	37.5	87.5	37.5	62.5	0	75.0
Forest cover	Climate change	9	44.4	44.4	11.1	33.3	22.2	22.	22.2	44.4
	Fire	28	32.1	32.1	7.1	21.4	14.3	10.7	7.1	17.9
	Land use	15	13.3	13.3	6.7	13.3	6.7	6.7	0.0	6.7

Note

Observations are individual occurrences of response/driver combinations. For details on the processes considered see Table 1.

#### 3.4.2 Legacy processes

Simulations of processes creating disturbance legacies were rare in the studies investigated (Table 4). Remaining live trees, indicating tolerance to disturbance, were the most common legacy implemented in models (28%). Twenty‐two percent of models differentiated between adult trees and saplings/seedlings in terms of susceptibility to disturbances. Seed banks were only simulated in a small number of studies investigating fire, despite the important role of seed banks in the recovery after fire in many ecosystems (Enright et al., 2015; Johnstone et al., 2010). We note, however, that serotiny (reported in the previous section on regeneration processes) equals the inclusion of a canopy seed bank. Approximately 12% of the model applications included some form of stress legacy, that is, where the model is able to simulate the compounding effect of stressors (such as drought) over multiple years, for example through a continuous simulation of carbohydrate reserves in trees (Hansen et al., 2018; McDowell et al., 2013).

**TABLE 4:** The explicit consideration of legacy processes in models used to simulate forest resilience, parsed by different combinations of resilience of what and resilience to what for the most commonly occurring combinations

		n	Legacies included (%)	Legacy processes			
				Disturbance tolerance (%)	Susceptibility by age (%)	Seed bank (%)	Persistent stress (%)
All model applications		128	33.6	28.1	21.8	6.3	11.7
**Of what**	**To what**						
Forest functioning	Climate change	21	38.1	33.3	19.0	4.8	14.3
	Fire	9	88.9	88.9	44.4	0	33.3
	Land use	10	80	70	30.0	0	20
Forest structure	Climate change	16	43.8	37.5	18.8	6.3	25
	Fire	14	50.0	42.9	35.7	0	28.6
	Land use	13	69.2	61.5	38.5	7.7	23.1
Forest composition	Climate change	14	57.1	57.1	35.7	14.3	28.6
	Fire	15	60.0	60.0	53.3	6.7	20
	Land use	8	87.5	87.5	50.0	12.5	25
Forest cover	Climate change	9	44.4	44.4	33.3	33.3	11.1
	Fire	28	21.4	21.4	28.6	10.7	3.6
	Land use	15	6.7	6.7	6.7	0	0

Note

Observations are individual occurrences of response/driver combinations. For details on the processes considered see Table 1.

#### 3.4.3 Soil processes

Soil processes related to forest resilience were more frequently included in models than regeneration and legacy processes (Table 5). Slightly less than half of the analysed cases considered water availability explicitly. However, water availability was often the only soil‐related process. Only around 20% of the applications considered a dynamic representation of the nitrogen cycle. Availability of other nutrients and their effect on ecological resilience were considered only rarely (< 1%, Supporting Information Material S3). Likewise, erosion processes, which influence forest resilience in some systems (Flores et al., 2019), were considered very rarely. Models assessing resilience of forest functioning had the most detailed representation of soil processes across all response indicators. Models that were applied to study the resilience to climate change tended to have a more complex representation of soil processes compared to investigations of other drivers.

**TABLE 5:** The explicit consideration of soil processes in models used to simulate forest resilience, parsed by different combinations of resilience of what and resilience to what for the most commonly occurring combinations

		n	Soil included (%)	Soil processes		
				Water availability (%)	Erosion (%)	Nitrogen cycle (%)
All model applications		128	46.1	46.8	3.9	18.75
**Of what**	**To what**					
Forest functioning	Climate change	21	76.2	85.7	0.0	38.1
	Fire	9	66.7	77.8	0.0	66.7
	Land use	10	80.0	70.0	20.0	30.0
Forest structure	Climate change	16	62.5	68.8	0.0	31.2
	Fire	14	42.9	57.1	7.1	42.9
	Land use	13	53.8	61.5	0.0	30.8
Forest composition	Climate change	14	71.4	92.9	0.0	57.1
	Fire	15	33.3	53.3	0.0	33.3
	Land use	8	62.5	75	0.0	50.0
Forest cover	Climate change	9	66.7	55.6	0.0	11.1
	Fire	28	17.9	21.4	0.0	7.1
	Land use	15	13.3	6.7	6.7	0.0

Note

Observations are individual occurrences of response/driver combinations. For details on the processes considered see Table 1.

## 4 DISCUSSION

Human‐caused climate change is challenging the resilience of forests. Developing adaptation strategies to mitigate these changes will require understanding of how multiple processes interact to shape resilience. Simulation models are a promising tool because they allow the exploration of a more complete set of compounding factors than field experiments and facilitate the analysis of outcomes over long time periods. However, in our review of the simulation models used for modelling forest resilience, we found that few were explicitly designed for that purpose and many relevant resilience processes are currently not well represented in models. Thus, we call for a new wave of model development that leverages expanding process understanding and data availability (e.g., from remote sensing) as well as growing computational resources.

While we here present the first comprehensive synthesis of models for forest resilience, our analysis has some limitations. One challenge in identifying relevant literature was the ambiguous use of the term resilience. On the one hand it is often used in abstracts and keywords of studies that do not actually investigate resilience based on commonly used resilience definitions. On the other hand, some studies that explicitly deal with modelling critical transitions and alternative stable states of ecosystems do not actually use the term resilience in their title and keywords. We addressed these challenges using multiple alternative keywords and carefully checking the studies we found for relevance before starting the in‐depth review process. The issue also underlines, however, that a more consistent and concise application of the resilience terminology in the literature would be desirable. Furthermore, the concept of resilience is intrinsically connected to concepts such as stability, vulnerability and persistence (Grimm & Wissel, 1997). Studies may address similar questions, but choose a different conceptual framework. Thus, some studies that address generally similar issues were excluded from our analysis due to our set of keywords. We focused on resilience because conceptual advances have been rapid and applications of resilience are growing both in the peer‐reviewed literature and society (Ratajczak et al., 2018; Selles & Rissman, 2020). Notwithstanding the challenges in identifying the relevant subset of the literature, important insights emerge from our review. We discuss these insights in the following sections, first focusing on the perspective of model users (Section 4.1) and then discussing issues of relevance for model developers and charting a path forward (Section 4.2).

### 4.1 Lessons learned for assessing forest resilience

A wide variety of resilience questions can be successfully tackled using models, as shown by the large number of driver‐response combinations found in the literature. Furthermore, many studies investigated the ecosystem response to multiple drivers (e.g., Batllori et al., 2017 for fire and drought, and Lucash et al., 2017 with LANDIS‐II for climate change, wind and forest management), which highlights a key strength of simulation models, that is, the ability to interactively consider a broad set of simultaneously changing factors. Models can also account for important feedbacks (for example between forest structure, species composition, and disturbances) that play a critical role in forest resilience (Flores et al., 2019; Staal et al., 2015).

Spatial and temporal extent of studies in our review varied widely. As spatio‐temporal extent is primarily defined by the specific research question being addressed, this finding underlines that models can provide inference across a wide range of applications. With regard to the temporal dimension, the long simulation durations (with a median of 200 years across all reviewed studies, and some studies extending over several thousands of simulated years, e.g., Bauch et al., 2016; Wild & Winkler, 2008) are a characteristic that sets simulation‐based studies apart from experimental and observational studies. A recent review of 89 drought experiments in terrestrial ecosystems found that 80% of drought treatments were less than 5 years in duration (Hoover et al., 2018). The ability to efficiently address centennial time‐scales makes models prime tools for assessing resilience questions that go beyond the analysis of individual disturbance events and rather focus on disturbance regimes (and changes thereof; e.g., Kitzberger et al., 2012, with SELES; Hudiburg et al., 2017, with DayCent). While models are a useful tool for simulating extensive time periods, long simulation time can also lead to unrealistic model behaviour due to compounding errors. Careful model evaluation (Oreskes et al., 1994) and the testing of simulations against a wide variety of observed spatial and temporal patterns (Grimm et al., 2005) are thus of particular importance to ensure the utility of long‐term simulations.

Our analysis shows that different types of model can provide unique insights on resilience, depending on which type of drivers and responses are of relevance in a given study system. When studying forest cover as the response variable of resilience, for instance, relatively simple models, such as sets of differential equations with varying levels of parametrization, were successfully applied to study transitions between forest and savanna (De Michele & Accatino, 2014; Tredennick & Hanan, 2015). However, when resilience of forest composition, structure and functioning is investigated, landscape models are powerful tools due to their spatially explicit nature and their ability to accommodate complex ecological processes (Scheller & Mladenoff, 2007; Shifley et al., 2017). This implies that more complex models are needed to simulate processes that lead to changes in composition, structure and functioning, whereas the consideration of basic demographic processes (establishment and mortality) in models is enough to reproduce the dynamics of forest cover. Indeed, most of the models that assessed ecosystem functions were landscape models or DGVMs. While choice of model is influenced by multiple considerations of suitability and availability, our review provides a starting point for considering potentially appropriate model families for studying specific questions of forest resilience (Figure 3).

There is no one best model type for a certain resilience question, as illustrated by the wide range of studies addressing forest resilience to fire. Most frequently applied are landscape models, in which spatial processes such as fire spread and seed dispersal into burnt areas can be simulated (e.g., Loudermilk et al., 2017, with LANDIS‐II; Keane et al., 2019, with FireBGCv2). However, the second most frequently used model type for studying the resilience to fire is analytical or conceptual models, where fire is generally represented aspatially and with simplified computation of tree mortality. Therefore, model choice depends strongly on the drivers and responses being simulated. We thus advocate for an approach where the appropriate model is chosen based on its ability to simulate the relevant mechanisms of resilience in a given context and study system (e.g., Hansen et al., 2018, with iLand).

### 4.2 Lessons learned for modelling forest resilience

We found a large gap between the processes that are considered to be important mechanisms of resilience in the theoretical and empirical literature (e.g., Flores et al., 2019; Johnstone et al., 2016; Martínez‐Vilalta & Lloret, 2016; von Oheimb et al., 2014) and the explicit consideration of these processes in models used to study forest resilience. In other words, processes deemed relevant for resilience (e.g., seed banks, soil erosion and nutrient cycling) are often not included in models. As a result, many models are not yet capable of comprehensively testing which theoretically important processes may underpin future forest resilience. Another possible outcome of missing resilience processes in models could be a systematic underestimation of resilience in simulation studies.

Process representation varies with the spatial scale of models. Landscape models (e.g., iLand, LANDIS‐II) are designed to simulate forests on relatively small spatial domains and are thus well suited to simulate critical fine‐scale processes and spatial dynamics. Indeed, our analyses revealed that several landscape models are able to capture a large number of relevant forest resilience mechanisms. Conversely, DGVMs and Earth system models that operate across continental to global domains and are successfully used to simulate questions of biome shifts and forest die‐back (Gonzalez et al., 2010; Higgins & Scheiter, 2012) are just beginning to represent forest demography with sufficient detail to explore questions of resilience (Fisher et al., 2018; Massoud et al., 2019; U.S. DOE, 2018).

While many types of models can increasingly capture aspects of resilience, most were not designed explicitly for this purpose. Thus, our analysis underscores the need for a new round of model development (Box 2). The resilience processes highlighted in our review (Tables 3, 4, 5, Supporting Information Materials S2 and S3) can provide a valuable starting point for such a resilience‐focused development of simulation models. While for many of these processes examples of how to model them already exist in the literature, some processes of resilience, such as plant trait adaptation (both local adaptation within populations as well as adaptation over time, such as acclimation processes) remain widely neglected in current models (Longo et al., 2018; Nitschke & Innes, 2008, but see Scheiter et al., 2013 for a model including adaptive trait combinations).

Box 2 **Future directions**

- The models currently in use for simulating resilience have often been developed for other purposes and are not fully capturing relevant processes of forest resilience.
- A new wave of model development is needed, especially focusing on processes not yet well‐represented in models (e.g., nutrient cycling, plant trait adaptation, tree regeneration).
- New empirical and experimental studies can contribute to model development by specifically targeting gaps in process understanding. This inter alia requires that the interactions between the model development and experimental/empirical communities are strengthened.

Here it should be noted that not all of the processes considered here are necessarily relevant for all study systems. There are, for instance, no serotinous tree species in Central Europe, which is why this process – important for the resilience of forests to fire in other areas of the world (Enright et al., 2015; Johnstone et al., 2016) – is not included in models applied in this region. While we designed our catalogue of model processes based on literature and tried to make it broadly applicable, there are likely many more processes that are relevant to resilience research, depending on the study system and questions asked. Our catalogue of processes does therefore not claim completeness (but see Supporting Information Materials S2 and S3 for an overview of the full set of processes we investigated, not all of which are analysed in depth here) and the separation into three categories of resilience processes is not always clear‐cut (e.g., we addressed seed banks as legacies, but serotiny and resprouting in the regeneration category).

There may also be processes that are not yet understood well enough to be modelled, highlighting the need for further experimental and empirical research (e.g., adaptation/plasticity of plant functional traits, Christmas et al., 2016). Authors frequently mention processes they consider relevant to their question and study system, but that have not been implemented in the applied model. These processes include, for instance, nutrient cycles (Bond‐Lamberty et al., 2015), as well as the effect of CO_2_ fertilization (Bagdon & Huang, 2014; Longo et al., 2018; Lucash et al., 2017), despite growing evidence that these processes are important for simulating vegetation dynamics (Hickler et al., 2015; Rammig et al., 2010; Reyer et al., 2014). Targeted model comparison experiments – applying models with different levels of mechanistic detail and differing implementations of processes to the same driver data – could shed more light on the uncertainties originating from representing processes in different ways or omitting them from models entirely (Bugmann et al., 2019; Petter et al., 2020).

A new wave of model development also requires improved data on forest resilience and its underlying mechanisms. Multiple authors mention data availability as a key obstacle to implementing more process details in models (e.g., Lucash et al., 2017; Magnuszewski et al., 2015). This highlights that further empirical and experimental work is crucially needed for developing more robust simulations of forest resilience. Specifically, empirical and experimental studies that are explicitly designed to address gaps in models could yield valuable insights. Also remote sensing is increasingly used to study forest resilience (De Keersmaecker et al., 2014; Senf et al., 2019) and can serve as a valuable data source for model‐based studies, especially when addressing forest change across large spatial extents and in areas where data are sparse (Levine et al., 2016; Staal et al., 2018). In particular, increasing availability of new datasets could be leveraged to benchmark models and identify process uncertainties. This can in turn direct the design of new experiments that address processes underpinning resilience and feed back into model development (Dietze et al., 2018). In the context of increasing the mechanistic details included in models several authors also discuss the trade‐off between model complexity and the computational resources needed to run such increasingly complex models (e.g., Manoli et al., 2017; Mitra et al., 2015). This means that applying more complex models may come at the cost of reduced study periods, a smaller number of simulated replicates, or a narrower set of driver combinations being investigated. Even with steadily increasing computational resources, increasingly complex models could thus result in a reduced inferential potential in certain applications, highlighting that the trade‐offs that come with higher process detail should be explicitly considered (Loehle, 1990).

Anthropogenic climate and land use change are profoundly affecting forests, emphasizing the need to understand how these impacts will alter forest ecosystems. Models play an important role in understanding the drivers and scope of these changes and the responses of forest ecosystems. Thus, deliberately developed and applied models can make an important contribution to understanding and managing ecological resilience in a changing world. Our study presents a valuable framework for assessing which currently available models are appropriate for such questions and can act as a starting point for a new generation of model developers.

## BIOSKETCH

Katharina Albrich is a PhD student at the University of Life Sciences and Natural Resources supervised by Prof. Rupert Seidl. Her research focuses on the resilience of forests to climate change and disturbances.

## Supporting information

Material S1

Material S2

Material S3

## Data Availability

Data and code used in the analysis are available at https://doi.org/10.6084/m9.figshare.12958166

## References

[geb13197-bib-0001] Adger, W. N. (2005). Social‐ecological resilience to coastal disasters. Science, 309, 1036–1039. 10.1126/science.1112122 16099974

[geb13197-bib-0002] Albrich, K. , Rammer, W. , & Seidl, R. (2020). Climate change causes critical transitions and irreversible alterations of mountain forests. Global Change Biology, 26, 4013–4027. 10.1111/gcb.15118 32301569 PMC7317840

[geb13197-bib-0003] Allaire, J. J. , Gandrud, C. , Russell, K. , & Yetman, C. J. (2017). networkD3: D3 javascript network graphs from R. https://CRAN.R-project.org/package=networkD3

[geb13197-bib-0004] Anderegg, W. R. L. , Schwalm, C. , Biondi, F. , Camarero, J. J. , Koch, G. , Litvak, M. , Ogle, K. , Shaw, J. D. , Shevliakova, E. , Williams, A. P. , Wolf, A. , Ziaco, E. , & Pacala, S. (2015). Pervasive drought legacies in forest ecosystems and their implications for carbon cycle models. Science, 349, 528–532. 10.1126/science.aab1833 26228147

[geb13197-bib-0005] Bagdon, B. , & Huang, C.‐H. (2014). Carbon stocks and climate change: Management implications in northern Arizona ponderosa pine forests. Forests, 5, 620–642. 10.3390/f5040620

[geb13197-bib-0006] Batllori, E. , De Cáceres, M. , Brotons, L. , Ackerly, D. D. , Moritz, M. A. , & Lloret, F. (2017). Cumulative effects of fire and drought in Mediterranean ecosystems. Ecosphere, 8, e01906 10.1002/ecs2.1906

[geb13197-bib-0007] Bauch, C. T. , Sigdel, R. , Pharaon, J. , & Anand, M. (2016). Early warning signals of regime shifts in coupled human‐environment systems. Proceedings of the National Academy of Sciences USA, 113, 14560–14567. 10.1073/pnas.1604978113 PMC5187665 27815533

[geb13197-bib-0008] Bond‐Lamberty, B. , Fisk, J. P. , Holm, J. A. , Bailey, V. , Bohrer, G. , & Gough, C. M. (2015). Moderate forest disturbance as a stringent test for gap and big‐leaf models. Biogeosciences, 12, 513–526. 10.5194/bg-12-513-2015

[geb13197-bib-0009] Bone, C. , Moseley, C. , Vinyeta, K. , & Bixler, R. P. (2016). Employing resilience in the United States forest service. Land Use Policy, 52, 430–438. 10.1016/j.landusepol.2016.01.003

[geb13197-bib-0010] Brand, F. S. , & Jax, K. (2007). Focusing the meaning(s) of resilience: Resilience as a descriptive concept and a boundary object. Ecology and Society, 12, art23. 10.5751/ES-02029-120123

[geb13197-bib-0011] Bugmann, H. , Seidl, R. , Hartig, F. , Bohn, F. , Brůna, J. , Cailleret, M. , François, L. , Heinke, J. , Henrot, A.‐J. , Hickler, T. , Hülsmann, L. , Huth, A. , Jacquemin, I. , Kollas, C. , Lasch‐Born, P. , Lexer, M. J. , Merganič, J. , Merganičová, K. , Mette, T. , … Reyer, C. P. O. (2019). Tree mortality submodels drive simulated long‐term forest dynamics: Assessing 15 models from the stand to global scale. Ecosphere, 10, e02616 10.1002/ecs2.2616 PMC8609442 34853712

[geb13197-bib-0012] Carpenter, S. , Walker, B. , Anderies, J. M. , & Abel, N. (2001). From metaphor to measurement: Resilience of what to what? Ecosystems, 4, 765–781. 10.1007/s10021-001-0045-9

[geb13197-bib-0013] Charnley, S. , Spies, T. A. , Barros, A. M. G. , White, E. M. , & Olsen, K. A. (2017). Diversity in forest management to reduce wildfire losses: Implications for resilience. Ecology and Society, 22, art22. 10.5751/ES-08753-220122

[geb13197-bib-0014] Christmas, M. J. , Breed, M. F. , & Lowe, A. J. (2016). Constraints to and conservation implications for climate change adaptation in plants. Conservation Genetics, 17, 305–320. 10.1007/s10592-015-0782-5

[geb13197-bib-0015] De Keersmaecker, W. , Lhermitte, S. , Honnay, O. , Farifteh, J. , Somers, B. , & Coppin, P. (2014). How to measure ecosystem stability? An evaluation of the reliability of stability metrics based on remote sensing time series across the major global ecosystems. Global Change Biology, 20, 2149–2161. 10.1111/gcb.12495 24777443

[geb13197-bib-0016] De Michele, C. , & Accatino, F. (2014). Tree cover bimodality in savannas and forests emerging from the switching between two fire dynamics. PLoS ONE, 9, 1–7. 10.1371/journal.pone.0091195 PMC3963849 24663432

[geb13197-bib-0017] Dietze, M. C. , Fox, A. , Beck‐Johnson, L. M. , Betancourt, J. L. , Hooten, M. B. , Jarnevich, C. S. , Keitt, T. H. , Kenney, M. A. , Laney, C. M. , Larsen, L. G. , Loescher, H. W. , Lunch, C. K. , Pijanowski, B. C. , Randerson, J. T. , Read, E. K. , Tredennick, A. T. , Vargas, R. , Weathers, K. C. , & White, E. P. (2018). Iterative near‐term ecological forecasting: Needs, opportunities, and challenges. Proceedings of the National Academy of Sciences USA, 115, 1424–1432. 10.1073/pnas.1710231115 PMC5816139 29382745

[geb13197-bib-0018] Egli, L. , Weise, H. , Radchuk, V. , Seppelt, R. , & Grimm, V. (2018). Exploring resilience with agent‐based models: State of the art, knowledge gaps and recommendations for coping with multidimensionality. Ecological Complexity. 40 Part B, 1–7.

[geb13197-bib-0019] Enright, N. J. , Fontaine, J. B. , Bowman, D. M. J. S. , Bradstock, R. A. , & Williams, R. J. (2015). Interval squeeze: Altered fire regimes and demographic responses interact to threaten woody species persistence as climate changes. Frontiers in Ecology and the Environment, 13, 265–272. 10.1890/140231

[geb13197-bib-0020] Enright, N. J. , Fontaine, J. B. , Lamont, B. B. , Miller, B. P. , & Westcott, V. C. (2014). Resistance and resilience to changing climate and fire regime depend on plant functional traits. Journal of Ecology, 102, 1572–1581. 10.1111/1365-2745.12306

[geb13197-bib-0021] Fahey, R. T. , Stuart‐Haëntjens, E. J. , Gough, C. M. , De La Cruz, A. , Stockton, E. , Vogel, C. S. , & Curtis, P. S. (2016). Evaluating forest subcanopy response to moderate severity disturbance and contribution to ecosystem‐level productivity and resilience. Forest Ecology and Management, 376, 135–147. 10.1016/j.foreco.2016.06.001

[geb13197-bib-0022] Fisher, R. A. , Koven, C. D. , Anderegg, W. R. L. , Christoffersen, B. O. , Dietze, M. C. , Farrior, C. E. , Holm, J. A. , Hurtt, G. C. , Knox, R. G. , Lawrence, P. J. , Lichstein, J. W. , Longo, M. , Matheny, A. M. , Medvigy, D. , Muller‐Landau, H. C. , Powell, T. L. , Serbin, S. P. , Sato, H. , Shuman, J. K. , … Moorcroft, P. R. (2018). Vegetation demographics in Earth System Models: A review of progress and priorities. Global Change Biology, 24, 35–54. 10.1111/gcb.13910 28921829

[geb13197-bib-0023] Flores, B. M. , Staal, A. , Jakovac, C. C. , Hirota, M. , Holmgren, M. , & Oliveira, R. S. (2019). Soil erosion as a resilience drain in disturbed tropical forests. Plant and Soil, 450, 11–25. 10.1007/s11104-019-04097-8

[geb13197-bib-0024] Folke, C. (2006). Resilience: The emergence of a perspective for social–ecological systems analyses. Global Environmental Change, 16, 253–267. 10.1016/j.gloenvcha.2006.04.002

[geb13197-bib-0025] Frelich, L. E. , & Reich, P. B. (1999). Minireviews: Neighborhood effects, disturbance severity, and community stability in forests. Ecosystems, 2, 151–166. 10.1007/s100219900066

[geb13197-bib-0026] Gazol, A. , Camarero, J. J. , Anderegg, W. R. L. , & Vicente‐Serrano, S. M. (2017). Impacts of droughts on the growth resilience of Northern Hemisphere forests. Global Ecology and Biogeography, 26, 166–176. 10.1111/geb.12526

[geb13197-bib-0027] Gonzalez, P. , Neilson, R. P. , Lenihan, J. M. , & Drapek, R. J. (2010). Global patterns in the vulnerability of ecosystems to vegetation shifts due to climate change. Global Ecology and Biogeography, 19, 755–768. 10.1111/j.1466-8238.2010.00558.x

[geb13197-bib-0028] Grimm, V. , Revilla, E. , Berger, U. , Jeltsch, F. , Mooij, W. M. , Railsback, S. F. , Thulke, H.‐H. , Weiner, J. , Wiegand, T. , & DeAngelis, D. L. (2005). Pattern‐oriented modeling of agent‐based complex systems: Lessons from ecology. Science, 310, 987–991. 10.1126/science.1116681 16284171

[geb13197-bib-0029] Grimm, V. , & Wissel, C. (1997). Babel, or the ecological stability discussions: An inventory and analysis of terminology and a guide for avoiding confusion. Oecologia, 109, 323–334. 10.1007/s004420050090 28307528

[geb13197-bib-0030] Halofsky, J. S. , Halofsky, J. E. , Burcsu, T. , & Hemstrom, M. A. (2014). Dry forest resilience varies under simulated climate‐management scenarios in a central Oregon, USA landscape. Ecological Applications, 24, 1908–1925. 10.1890/13-1653.1 29185662

[geb13197-bib-0031] Hansen, W. D. , Braziunas, K. H. , Rammer, W. , Seidl, R. , & Turner, M. G. (2018). It takes a few to tango: Changing climate and fire regimes can cause regeneration failure of two subalpine conifers. Ecology, 99, 966–977. 10.1002/ecy.2181 29464688

[geb13197-bib-0032] Hickler, T. , Rammig, A. , & Werner, C. (2015). Modelling CO_2_ impacts on forest productivity. Current Forestry Reports, 1, 69–80. 10.1007/s40725-015-0014-8

[geb13197-bib-0033] Higgins, S. I. , & Scheiter, S. (2012). Atmospheric CO_2_ forces abrupt vegetation shifts locally, but not globally. Nature, 488, 209–212. 10.1038/nature11238 22763447

[geb13197-bib-0034] Holling, C. S. (1973). Resilience and stability of ecological systems. Annual Review of Ecology and Systematics, 4, 1–23. 10.1146/annurev.es.04.110173.000245

[geb13197-bib-0035] Holling, C. S. , & Gunderson, L. H. (2002). Resilience and adaptive cycles In Gunderson L. H. & Holling C. S. (Eds.), Panarchy: Understanding the transformations in human and natural systems (pp. 25–62). Island Press.

[geb13197-bib-0036] Hoover, D. L. , Wilcox, K. R. , & Young, K. E. (2018). Experimental droughts with rainout shelters: A methodological review. Ecosphere, 9, 1–14. 10.1002/ecs2.2088

[geb13197-bib-0037] Hudiburg, T. W. , Higuera, P. E. , & Hicke, J. A. (2017). Fire‐regime variability impacts forest carbon dynamics for centuries to millennia. Biogeosciences, 14, 3873–3882. 10.5194/bg-14-3873-2017

[geb13197-bib-0038] Johnstone, J. F. , Allen, C. D. , Franklin, J. F. , Frelich, L. E. , Harvey, B. J. , Higuera, P. E. , Mack, M. C. , Meentemeyer, R. K. , Metz, M. R. , Perry, G. L. W. , Schoennagel, T. , & Turner, M. G. (2016). Changing disturbance regimes, ecological memory, and forest resilience. Frontiers in Ecology and the Environment, 14, 369–378. 10.1002/fee.1311

[geb13197-bib-0039] Johnstone, J. F. , Chapin, F. S. , Hollingsworth, T. N. , Mack, M. C. , Romanovsky, V. , & Turetsky, M. (2010). Fire, climate change, and forest resilience in interior Alaska. Canadian Journal of Forest Research, 40, 1302–1312.

[geb13197-bib-0040] Keane, R. E. , Gray, K. , Davis, B. , Holsinger, L. M. , & Loehman, R. (2019). Evaluating ecological resilience across wildfire suppression levels under climate and fuel treatment scenarios using landscape simulation modelling. International Journal of Wildland Fire, 28, 533 10.1071/WF19015

[geb13197-bib-0041] Keane, R. E. , Hessburg, P. F. , Landres, P. B. , & Swanson, F. J. (2009). The use of historical range and variability (HRV) in landscape management. Forest Ecology and Management, 258, 1025–1037. 10.1016/j.foreco.2009.05.035

[geb13197-bib-0042] Keenan, R. J. (2015). Climate change impacts and adaptation in forest management: A review. Annals of Forest Science, 72, 145–167. 10.1007/s13595-014-0446-5

[geb13197-bib-0043] Kitzberger, T. , Aráoz, E. , Gowda, J. H. , Mermoz, M. , & Morales, J. M. (2012). Decreases in fire spread probability with forest age promotes alternative community states, reduced resilience to climate variability and large fire regime shifts. Ecosystems, 15, 97–112. 10.1007/s10021-011-9494-y

[geb13197-bib-0044] Levine, N. M. , Zhang, K. , Longo, M. , Baccini, A. , Phillips, O. L. , Lewis, S. L. , Alvarez‐Dávila, E. , Segalin de Andrade, A. C. , Brienen, R. J. W. , Erwin, T. L. , Feldpausch, T. R. , Monteagudo Mendoza, A. L. , Nuñez Vargas, P. , Prieto, A. , Silva‐Espejo, J. E. , Malhi, Y. , & Moorcroft, P. R. (2016). Ecosystem heterogeneity determines the ecological resilience of the Amazon to climate change. Proceedings of the National Academy of Sciences USA, 113, 793–797. 10.1073/pnas.1511344112 PMC4725538 26711984

[geb13197-bib-0045] Loehle, C. (1990). A guide to increased creativity in research: Inspiration or perspiration? BioScience, 40, 123–129. 10.2307/1311345

[geb13197-bib-0046] Longo, M. , Knox, R. G. , Levine, N. M. , Alves, L. F. , Bonal, D. , Camargo, P. B. , Fitzjarrald, D. R. , Hayek, M. N. , Restrepo‐Coupe, N. , Saleska, S. R. , da Silva, R. , Stark, S. C. , Tapajós, R. P. , Wiedemann, K. T. , Zhang, K. , Wofsy, S. C. , & Moorcroft, P. R. (2018). Ecosystem heterogeneity and diversity mitigate Amazon forest resilience to frequent extreme droughts. New Phytologist, 219, 914–931. 10.1111/nph.15185 29786858

[geb13197-bib-0047] Loudermilk, E. L. , Scheller, R. M. , Weisberg, P. J. , & Kretchun, A. (2017). Bending the carbon curve: Fire management for carbon resilience under climate change. Landscape Ecology, 32, 1461–1472. 10.1007/s10980-016-0447-x

[geb13197-bib-0048] Lucash, M. S. , Scheller, R. M. , J. Gustafson, E. , & R. Sturtevant, B. (2017). Spatial resilience of forested landscapes under climate change and management. Landscape Ecology, 32, 953–969. 10.1007/s10980-017-0501-3

[geb13197-bib-0049] Magnuszewski, P. , Ostasiewicz, K. , Chazdon, R. , Salk, C. , Pajak, M. , Sendzimir, J. , & Andersson, K. (2015). Resilience and alternative stable states of tropical forest landscapes under shifting cultivation regimes. PLoS ONE, 10, e0137497 10.1371/journal.pone.0137497 26406907 PMC4584006

[geb13197-bib-0050] Manoli, G. , Huang, C. W. , Bonetti, S. , Domec, J. C. , Marani, M. , & Katul, G. (2017). Competition for light and water in a coupled soil‐plant system. Advances in Water Resources, 108, 216–230. 10.1016/j.advwatres.2017.08.004

[geb13197-bib-0051] Martínez‐Vilalta, J. , & Lloret, F. (2016). Drought‐induced vegetation shifts in terrestrial ecosystems: The key role of regeneration dynamics. Global and Planetary Change, 144, 94–108. 10.1016/j.gloplacha.2016.07.009

[geb13197-bib-0052] Massoud, E. C. , Xu, C. , Fisher, R. A. , Knox, R. G. , Walker, A. P. , Serbin, S. P. , Christoffersen, B. O. , Holm, J. A. , Kueppers, L. M. , Ricciuto, D. M. , Wei, L. , Johnson, D. J. , Chambers, J. Q. , Koven, C. D. , McDowell, N. G. , & Vrugt, J. A. (2019). Identification of key parameters controlling demographically structured vegetation dynamics in a land surface model: CLM4.5(FATES). Geoscientific Model Development, 12, 4133–4164. 10.5194/gmd-12-4133-2019

[geb13197-bib-0053] McDowell, N. G. , Allen, C. D. , Anderson‐Teixeira, K. , Aukema, B. H. , Bond‐Lamberty, B. , Chini, L. , Clark, J. S. , Dietze, M. , Grossiord, C. , Hanbury‐Brown, A. , Hurtt, G. C. , Jackson, R. B. , Johnson, D. J. , Kueppers, L. , Lichstein, J. W. , Ogle, K. , Poulter, B. , Pugh, T. A. M. , Seidl, R. , … Xu, C. (2020). Pervasive shifts in forest dynamics in a changing world. Science, 368, 1–10. 10.1126/science.aaz9463 32467364

[geb13197-bib-0054] McDowell, N. G. , Fisher, R. A. , Xu, C. , Domec, J. C. , Hölttä, T. , Mackay, D. S. , Sperry, J. S. , Boutz, A. , Dickman, L. , Gehres, N. , Limousin, J. M. , Macalady, A. , Martínez‐Vilalta, J. , Mencuccini, M. , Plaut, J. A. , Ogée, J. , Pangle, R. E. , Rasse, D. P. , Ryan, M. G. , … Pockman, W. T. (2013). Evaluating theories of drought‐induced vegetation mortality using a multimodel‐experiment framework. New Phytologist, 200, 304–321. 10.1111/nph.12465 24004027

[geb13197-bib-0055] McDowell, N. , Pockman, W. T. , Allen, C. D. , Breshears, D. D. , Cobb, N. , Kolb, T. , Plaut, J. , Sperry, J. , West, A. , Williams, D. G. , & Yepez, E. A. (2008). Mechanisms of plant survival and mortality during drought: Why do some plants survive while others succumb to drought? New Phytologist, 178, 719–739. 10.1111/j.1469-8137.2008.02436.x 18422905

[geb13197-bib-0056] Millar, C. I. , Stephenson, N. L. , & Stephens, S. L. (2007). Climate change and forests of the future: Managing in the face of uncertainty. Ecological Applications, 17, 2145–2151. 10.1890/06-1715.1 18213958

[geb13197-bib-0057] Mitra, C. , Kurths, J. , & Donner, R. V. (2015). An integrative quantifier of multistability in complex systems based on ecological resilience. Scientific Reports, 5, 16196 10.1038/srep16196 26537459 PMC4633666

[geb13197-bib-0058] Nikinmaa, L. , Lindner, M. , Cantarello, E. , Jump, A. S. , Seidl, R. , Winkel, G. , & Muys, B. (2020). Reviewing the use of resilience concepts in forest sciences. Current Forestry Reports, 6, 61–80. 10.1007/s40725-020-00110-x PMC7612878 35747899

[geb13197-bib-0059] Nitschke, C. R. , & Innes, J. L. (2008). A tree and climate assessment tool for modelling ecosystem response to climate change. Ecological Modelling, 210, 263–277. 10.1016/j.ecolmodel.2007.07.026

[geb13197-bib-0060] Olson, D. M. , Dinerstein, E. , Wikramanayake, E. D. , Burgess, N. D. , Powell, G. V. N. , Underwood, E. C. , D'amico, J. A. , Itoua, I. , Strand, H. E. , Morrison, J. C. , Loucks, C. J. , Allnutt, T. F. , Ricketts, T. H. , Kura, Y. , Lamoreux, J. F. , Wettengel, W. W. , Hedao, P. , & Kassem, K. R. (2001). Terrestrial ecoregions of the world: A new map of life on earth. BioScience, 51, 933–938. 10.1641/0006-3568(2001)051[0933:TEOTWA]2.0.CO;2

[geb13197-bib-0061] Oreskes, N. , Shrader‐Frechette, K. , & Belitz, K. (1994). Verification, validation, and confirmation of numerical models in the earth sciences. Science, 263, 641–646. 10.1126/science.263.5147.641 17747657

[geb13197-bib-0062] Petter, G. , Mairota, P. , Albrich, K. , Bebi, P. , Brůna, J. , Bugmann, H. , Haffenden, A. , Scheller, R. M. , Schmatz, D. R. , Seidl, R. , Speich, M. , Vacchiano, G. , & Lischke, H. (2020). How robust are future projections of forest landscape dynamics? Insights from a systematic comparison of four forest landscape models. Environmental Modelling & Software, 134, 104844 10.1016/j.envsoft.2020.104844

[geb13197-bib-0063] Pimm, S. L. (1984). The complexity and stability of ecosystems. Nature, 307, 321–326. 10.1038/307321a0

[geb13197-bib-0064] R Core Team (2019). R: A language and environment for statistical computing, Vienna, Austria: R Foundation for Statistical Computing.

[geb13197-bib-0092] Rammig, A. , Jupp, T. , Thonicke, K. , Tietjen, B. , Heinke, J. , Ostberg, S. , Lucht, W. , Cramer, W. , & Cox, P. (2010). Estimating the risk of Amazonian forest dieback. New Phytologist, 187, 694–706. 10.1111/j.1469-8137.2010.03318.x 20553387

[geb13197-bib-0065] Ratajczak, Z. , Carpenter, S. R. , Ives, A. R. , Kucharik, C. J. , Ramiadantsoa, T. , Stegner, M. A. , Williams, J. W. , Zhang, J. , & Turner, M. G. (2018). Abrupt change in ecological systems: Inference and diagnosis. Trends in Ecology and Evolution, 33, 513–526. 10.1016/j.tree.2018.04.013 29784428

[geb13197-bib-0066] Reyer, C. , Lasch‐Born, P. , Suckow, F. , Gutsch, M. , Murawski, A. , & Pilz, T. (2014). Projections of regional changes in forest net primary productivity for different tree species in Europe driven by climate change and carbon dioxide. Annals of Forest Science, 71, 211–225. 10.1007/s13595-013-0306-8

[geb13197-bib-0067] Rist, L. , & Moen, J. (2013). Sustainability in forest management and a new role for resilience thinking. Forest Ecology and Management, 310, 416–427. 10.1016/j.foreco.2013.08.033

[geb13197-bib-0068] Scheffer, M. , Carpenter, S. R. , Dakos, V. , & van Nes, E. H. (2015). Generic indicators of ecological resilience: Inferring the chance of a critical transition. Annual Review of Ecology, Evolution, and Systematics, 46, 145–167. 10.1146/annurev-ecolsys-112414-054242

[geb13197-bib-0069] Scheiter, S. , Langan, L. , & Higgins, S. I. (2013). Next‐generation dynamic global vegetation models: Learning from community ecology. New Phytologist, 198, 957–969. 10.1111/nph.12210 23496172

[geb13197-bib-0070] Scheller, R. M. , & Mladenoff, D. J. (2007). An ecological classification of forest landscape simulation models: Tools and strategies for understanding broad‐scale forested ecosystems. Landscape Ecology, 22, 491–505. 10.1007/s10980-006-9048-4

[geb13197-bib-0071] Scholze, M. , Knorr, W. , Arnell, N. W. , & Prentice, I. C. (2006). A climate‐change risk analysis for world ecosystems. Proceedings of the National Academy of Sciences USA, 103, 13116–13120. 10.1073/pnas.0601816103 PMC1559762 16924112

[geb13197-bib-0072] Schröder, A. , Persson, L. , & De Roos, A. M. (2005). Direct experimental evidence for alternative stable states: A review. Oikos, 110, 3–19. 10.1111/j.0030-1299.2005.13962.x

[geb13197-bib-0073] Seidl, R. , Rammer, W. , & Spies, T. A. (2014). Disturbance legacies increase the resilience of forest ecosystem structure, composition, and functioning. Ecological Applications, 24, 2063–2077. 10.1890/14-0255.1 27053913 PMC4820056

[geb13197-bib-0074] Seidl, R. , Spies, T. A. , Peterson, D. L. , Stephens, S. L. , & Hicke, J. A. (2016). Searching for resilience: Addressing the impacts of changing disturbance regimes on forest ecosystem services. Journal of Applied Ecology, 53, 120–129. 10.1111/1365-2664.12511 PMC4780065 26966320

[geb13197-bib-0075] Seidl, R. , Vigl, F. , Rössler, G. , Neumann, M. , & Rammer, W. (2017). Assessing the resilience of Norway spruce forests through a model‐based reanalysis of thinning trials. Forest Ecology and Management, 388, 3–12. 10.1016/j.foreco.2016.11.030 28860674 PMC5572630

[geb13197-bib-0076] Selles, O. A. , & Rissman, A. R. (2020). Content analysis of resilience in forest fire science and management. Land Use Policy, 94, 104483 10.1016/j.landusepol.2020.104483

[geb13197-bib-0077] Senf, C. , Müller, J. , & Seidl, R. (2019). Post‐disturbance recovery of forest cover and tree height differ with management in Central Europe. Landscape Ecology, 34, 2837–2850. 10.1007/s10980-019-00921-9

[geb13197-bib-0078] Shifley, S. R. , He, H. S. , Lischke, H. , Wang, W. J. , Jin, W. , Gustafson, E. J. , Thompson, J. R. , Thompson, F. R. , Dijak, W. D. , & Yang, J. (2017). The past and future of modeling forest dynamics: From growth and yield curves to forest landscape models. Landscape Ecology, 32(7), 1307–1325. 10.1007/s10980-017-0540-9

[geb13197-bib-0079] Shuman, J. K. , Shugart, H. H. , & O’Halloran, T. L. (2011). Sensitivity of Siberian larch forests to climate change. Global Change Biology, 17, 2370–2384. 10.1111/j.1365-2486.2011.02417.x

[geb13197-bib-0080] Staal, A. , Dekker, S. C. , Hirota, M. , & van Nes, E. H. (2015). Synergistic effects of drought and deforestation on the resilience of the south‐eastern Amazon rainforest. Ecological Complexity, 22, 65–75. 10.1016/j.ecocom.2015.01.003

[geb13197-bib-0081] Staal, A. , van Nes, E. H. , Hantson, S. , Holmgren, M. , Dekker, S. C. , Pueyo, S. , Xu, C. , & Scheffer, M. (2018). Resilience of tropical tree cover: The roles of climate, fire and herbivory. Global Change Biology, 24(11), 5096–5109. 10.1111/gcb.14408 30058246

[geb13197-bib-0082] Standish, R. J. , Hobbs, R. J. , Mayfield, M. M. , Bestelmeyer, B. T. , Suding, K. N. , Battaglia, L. L. , Eviner, V. , Hawkes, C. V. , Temperton, V. M. , Cramer, V. A. , Harris, J. A. , Funk, J. L. , & Thomas, P. A. (2014). Resilience in ecology: Abstraction, distraction, or where the action is? Biological Conservation, 177, 43–51. 10.1016/j.biocon.2014.06.008

[geb13197-bib-0083] Tredennick, A. T. , & Hanan, N. P. (2015). Effects of tree harvest on the stable‐state dynamics of savanna and forest. The American Naturalist, 185, E153–E165. 10.1086/680475 25905514

[geb13197-bib-0084] Trumbore, S. , Brando, P. , & Hartmann, H. (2015). Forest health and global change. Science, 349, 814–818. 10.1126/science.aac6759 26293952

[geb13197-bib-0085] U.S. DOE (2018). Disturbance and vegetation dynamics in earth system models; Workshop report, DOE/SC‐0196, Washington, DC: Office of Biological and Environmental Research and U.S. Department of Energy Office of Science.

[geb13197-bib-0086] von Oheimb, G. , Härdtle, W. , Eckstein, D. , Engelke, H.‐H. , Hehnke, T. , Wagner, B. , & Fichtner, A. (2014). Does forest continuity enhance the resilience of trees to environmental change? PLoS ONE, 9, e113507 10.1371/journal.pone.0113507 25494042 PMC4262476

[geb13197-bib-0087] Walker, B. , Holling, C. S. , Carpenter, S. R. , & Kinzig, A. P. (2004). Resilience, adaptability and transformability in social‐ecological systems. Ecology and Society, 9, art5. 10.5751/ES-00650-090205

[geb13197-bib-0088] Wickham, H. (2007). Reshaping data with the reshape package. Journal of Statistical Software, 21, 1–20.

[geb13197-bib-0089] Wickham, H. (2016). ggplot2: Elegant graphics for data analysis. Springer Verlag.

[geb13197-bib-0090] Wickham, H. , François, R. , Henry, L. , & Müller, K. (2019). dplyr: A grammar of data manipulation. https://CRAN.R-project.org/package-dplyr

[geb13197-bib-0091] Wild, J. , & Winkler, E. (2008). Krummholz and grassland coexistence above the forest‐line in the Krkonoše Mountains: Grid‐based model of shrub dynamics. Ecological Modelling, 213, 293–307. 10.1016/j.ecolmodel.2007.12.013

