# Supplementary material for: Simulating forest resilience: A review
Source: Glob Ecol Biogeogr. 2020 Oct 8;29(12):2082–96. doi: 10.1111/geb.13197 (PMC7756463; doi:10.1111/geb.13197)
Supplement: Supplementary file 1 — Material S1 [file GEB-29-2082-s001.docx]

**Simulating forest resilience: a review**

**Supplementary Materials S1: Supplementary results**


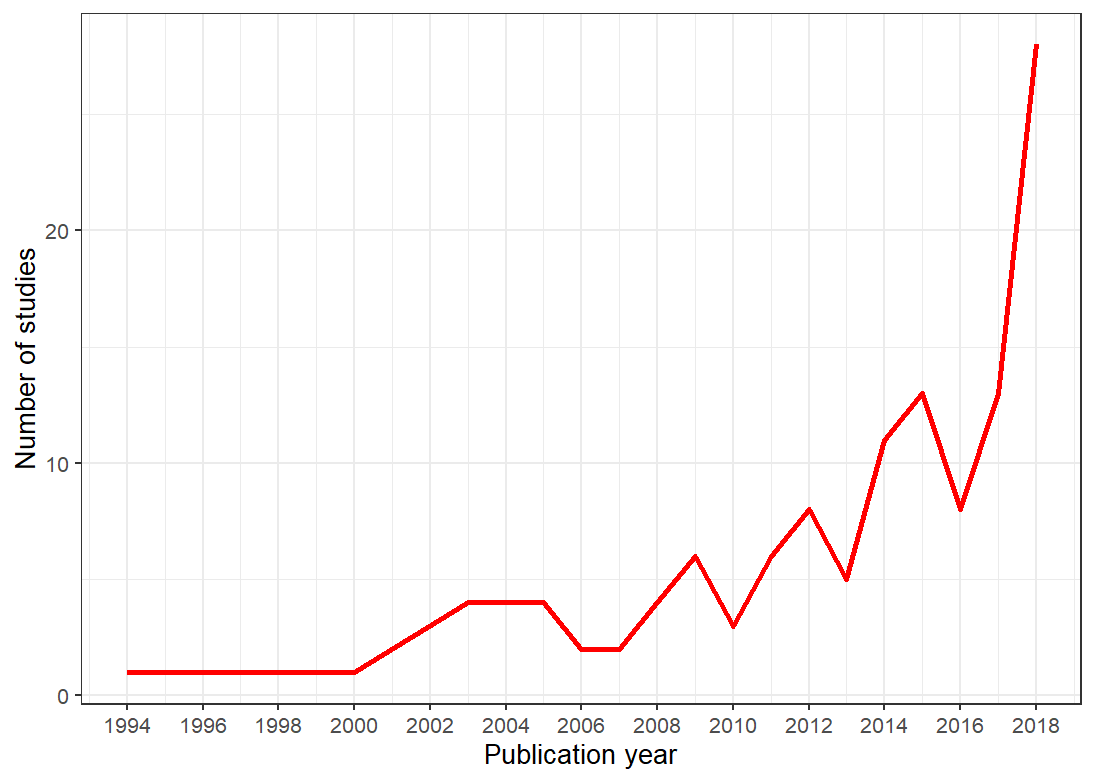


Figure S1.1: Publication of studies related to forest resilience modelling over time.

Figure S1.2: Drivers (resilience to what) commonly modelled together. Red stands for primarily anthropogenic drivers, blue for primarily non-anthropogenic.


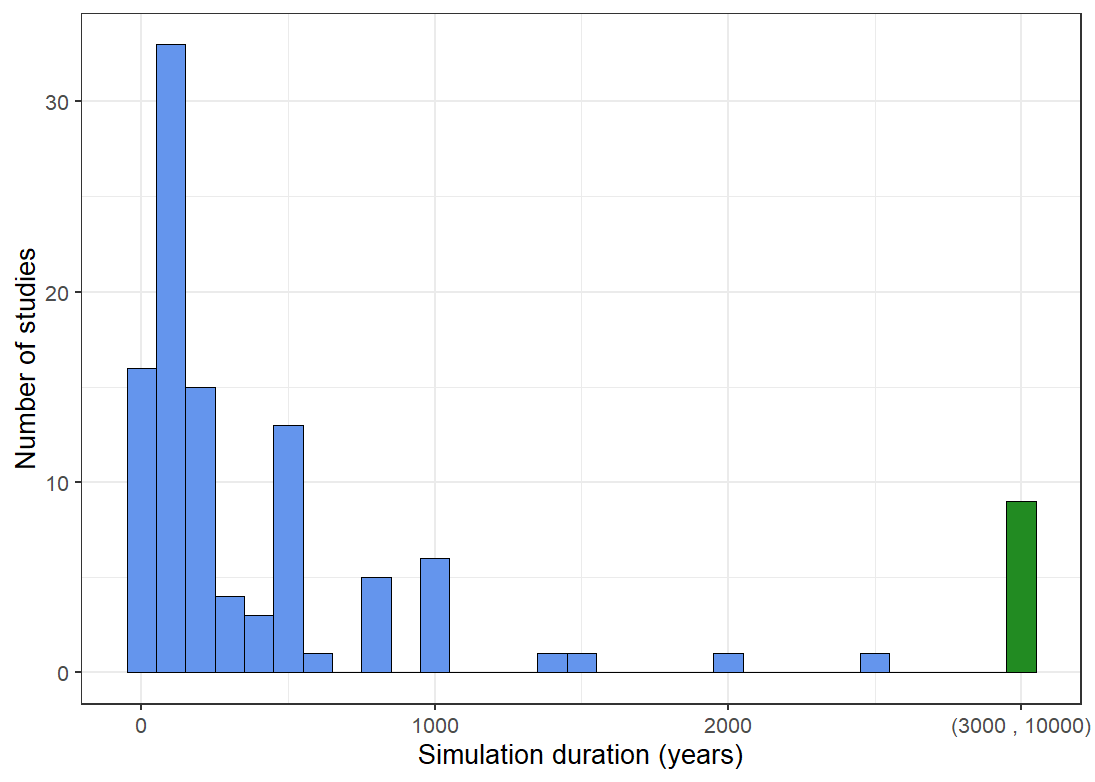


**Figure S1.3**: Histogram of simulation duration in years. Durations above 3000 years are binned into a single bin (maximum simulation duration was 10000 years).
