# Supplementary material for: Simulating forest resilience: A review
Source: Glob Ecol Biogeogr. 2020 Oct 8;29(12):2082–96. doi: 10.1111/geb.13197 (PMC7756463; doi:10.1111/geb.13197)
Supplement: Supplementary file 2 — Material S2 [file GEB-29-2082-s002.docx]

**Simulating forest resilience: a review**

**Supplementary Materials S2: Catalogue of questions assessed for each study with explanations**

**Meta-data**

- **Title of paper**
- **Name of first author**
- **Publication year**
- **doi**
- **use “resilience”: is the term resilience used in the paper**
- **Multiple locations:** are there several discrete study sites (plots/stands/landscapes) mentioned in the study
- **Continent:** continent on which study site is located (chose multiple if more than one location)
- **Country:** country where study site is located (write all if multiple)
- **Biome:** select most closely matching biome (chose multiple more than one location)
- **Dominant forest type:** select from coniferous/broadleaved/mixed (chose multiple more than one location with different forest types)
- **Ecosystem types:** briefly describe ecosystem types (including species) as they are given in the paper. Also include non-forest types if applicable

**Simulation model**

- **Model:** name of model + extensions used (“unnamed” if there is no specific name for the model)
- **Type of model (select):** choose the category which most closely fits the model described (population model, biogeochemical model, landscape model, dynamic global vegetation model, State and Transition model, empirical model, other)
- **Type of model (self-described):** how do the authors describe the model
- **Additional sources for model:** papers prominently cited which hold additional info about the model (for quick reference when needed)
- **Spatially explicit:** is the model spatially explicit and allows for spatial interactions?
- **Extent of study area**: in ha
- **Spatial grain:** in ha
- **Time step:** what is the smallest time step (highest temporal resolution) the model is operating on?
- **Longest Simulation duration:** how many years are simulated
- **Basic simulation entity:** what is the basic tree entity being modelled (trees, cohorts…)
- **Life forms other than trees (select):** which life forms other than trees (shrubs, herbs, grasses, other) are being modelled?
- **Sensitivity of lifeforms:** are life-forms sensitive to environmental surroundings (climate, substrate, competition) – list all life forms checked in the previous question and add “yes” if adaptive, “no” if not.
- **Sensitivity climate**: in particular, are trees sensitive to climate influences?
- **Growth included:** does the model include tree growth?
- **Mortality included:** is there a mortality process in the model?
- **Establishment included**: is there any establishment process in the model?
- **Competition included:** do trees compete for resources?
- **Stochastic:** are there any stochastic processes in the model?
- **Stochastic (describe processes):** where does stochasticity occur in the model?
- **Process-based:** is the model process-based?

**Resilience definition and quantification**

- **Definition of resilience:** select engineering (e.g. recovery is discussed) or ecological (e.g. alternative states are discussed) or other/undefined (if it fits neither of the other two categories.
- **Of what (select):** feature of the ecosystem the resilience of which is being investigated, select from general groups forest cover, forest structure, forest composition, forest functioning, ecosystem services, biodiversity
- **Of what (describe):** describe exactly what is being investigated (indicators)
- **To what (select):** wind, fire, insect, pathogen, drought, land use influence, other biotic, other abiotic, generic (no agent mentioned) and other
- **To what (describe):** describe the “to what” in more detail if needed
- **Multiple “of what”:** are there multiple “of what” in the study?
- **Multiple “to what”:** are there multiple “to what” in the study?
- **Interaction of different “to what” factors:** if multiple “to what” are investigated, are there interactions (e.g., climate change and natural disturbances)?
- **Interaction of different “to what” factors (describe):** which “to what” factors interact?
- **Impact of “to what”:** is the “to what” dynamically simulated, (i.e. impact depends on forest vegetation characteristics, etc.) or generic (i.e. killing all trees in a specified cell)?
- **Climate modelled:** is climate part of the model?
- **Climate variability:** is the climate static or is there variability?
- **Management implemented:** is forest management happening in the study?
- **Management considered as a disturbance?** is forest management seen as a disturbance?
- **Quantification of resilience:** which resilience metrics are employed (e.g. recovery time, difference in composition at two points in time,…)
- **Threshold:** is there a threshold in the response variable (as described by the authors)?
- **Alternative states:** do the authors describe alternative states?
- **Alternative states (describe):** which alternative states occur in the study system (e.g. conifer-dominated/broadleaf-dominated or forest/non-forest)?
- **Feedbacks:** are there any feedbacks mentioned?
- **Hysteresis:** does hysteresis occur in the transition between states?

**Processes of resilience: Regeneration:**

- **Natural regeneration included:** Is natural regeneration being modelled?
- **Multiple regeneration processes:** is regeneration expressed by multiple explicit processes (e.g. seed production, seed dispersal, germination, seedling survival) or in one process or probability of regeneration?
- **Regeneration processes mentioned**: if there are separate regeneration processes, which are these?
- **Reproductive maturity:** Do trees have to reach maturity before they can reproduce?
- **Masting:** does masting occur (annual fluctuations in seed availability)?
- **Serotiny:** does the model include serotiny for fire-adapted species
- **Resprouting**: are trees in the model able to resprout after disturbance
- **Distance to seed source:** Does distance to seed source influence regeneration success?
- **Substrate influence:** does the substrate (soil depth, nutrients…) influence regeneration success?
  **Climate influence:** is regeneration climate-sensitive?
- **Light availability:** does light availability influence regeneration success (e.g. by shading from canopy trees?)
- **Competition from ground vegetation**: does non-tree competition vegetation (grass, herbs, shrubs,…) influence regeneration success?
- **Herbivory/Biotic disturbances:** is regeneration success affected by herbivory?

**Processes of resilience: Legacies**

- **Disturbance tolerance:** do life trees remain in place after disturbance and act as seed source (rather than all trees in the disturbance affected area being killed)?
- **Susceptibility by age:** Does the age of trees (adult tree vs sapling) influence their environmental response/susceptibility to disturbance?
- **Seed banks:** does the model allow for seeds to be stored in seed banks (soil, canopy)?
- **Seedling banks**: can seedlings survive a disturbance or are they killed along with the adult trees?
- **Dead plant biomass:** does dead plant biomass stay behind after disturbance (and contribute to regeneration)?
- **Stress legacies:** are trees more susceptible to disturbances and other environmental pressures if they have experienced prior stress?
- **Adaptation/information legacies:** can trees adapt to environmental changes (e.g. by changing allocation patterns)?

**Process of resilience: Soil:**

- **Soil modelled:** are soil dynamics part of the model?
- **Spatial variation in soil:** are soil characteristics homogenous for the whole simulation area or is there spatial variation?
- **Water availability:** is water availability dynamically modelled?
- **Erosion:** does soil erosion happen in the model?
- **Nitrogen cycle:** is there are dynamic nitrogen cycle with feedbacks to plant-available nitrogen?
- **Other nutrient cycles:** are there other dynamic nutrient cycles with feedbacks to plant-available nutrients.

- **Other comments:** any other relevant features of the study.
- **Investigator:** who entered the study into the database?
